# Free Radical–Associated Gene Signature Predicts Survival in Sepsis Patients

**DOI:** 10.3390/ijms25084574

**Published:** 2024-04-22

**Authors:** Anlin Feng, Marissa D. Pokharel, Ying Liang, Wenli Ma, Saurabh Aggarwal, Stephen M. Black, Ting Wang

**Affiliations:** 1 Center for Translational Science, Florida International University, Port Saint Lucie, FL 34987, USA; 2 Department of Environmental Health Sciences, Florida International University, Miami, FL 33199, USA; 3 Department of Cellular and Molecular Medicine, Florida International University, Miami, FL 33199, USA

**Keywords:** molecular signature, ROS, prognosis, sepsis

## Abstract

Sepsis continues to overwhelm hospital systems with its high mortality rate and prevalence. A strategy to reduce the strain of sepsis on hospital systems is to develop a diagnostic/prognostic measure that identifies patients who are more susceptible to septic death. Current biomarkers fail to achieve this outcome, as they only have moderate diagnostic power and limited prognostic capabilities. Sepsis disrupts a multitude of pathways in many different organ systems, making the identification of a single powerful biomarker difficult to achieve. However, a common feature of many of these perturbed pathways is the increased generation of reactive oxygen species (ROS), which can alter gene expression, changes in which may precede the clinical manifestation of severe sepsis. Therefore, the aim of this study was to evaluate whether ROS-related circulating molecular signature can be used as a tool to predict sepsis survival. Here we created a ROS-related gene signature and used two Gene Expression Omnibus datasets from whole blood samples of septic patients to generate a 37-gene molecular signature that can predict survival of sepsis patients. Our results indicate that peripheral blood gene expression data can be used to predict the survival of sepsis patients by assessing the gene expression pattern of free radical–associated -related genes in patients, warranting further exploration.

## 1. Introduction

Sepsis is defined as life-threatening organ dysfunction caused by a dysregulated host response to infection [1]. Sepsis continues to devastate hospitals with its high mortality rate and prevalence. In the United States, sepsis is the most common cause of in-hospital deaths and is estimated to cost more than USD 24 billion annually [2,3]. Although the exact global burden of sepsis remains unknown, a 2020 meta-analysis study estimated that in 2017 there were 48.9 million cases of sepsis and 11.0 million sepsis-related deaths, representing approximately 20% of all global deaths [4]. Unfortunately, no effective therapies exist for sepsis, signaling the urgent need to diagnose septic cases early to begin appropriate antibiotic treatment and other preventative measures to prevent the onset of organ failure.

Further hindering the treatment and prevention of sepsis is the inability to differentiate severe septic patients who are at increased risk of death from milder cases of sepsis. A diagnostic measure to identify high-risk septic patients is critical, as this will alert healthcare providers to which patients need increased monitoring and implement more aggressive treatments or protocols in hopes of preventing septic shock/organ failure. Current biomarkers, such as C-reactive protein, lactate, and procalcitonin only have moderate diagnostic power and provide very limited prognostic capabilities. For example, procalcitonin has both low sensitivity and specificity for differentiating sepsis from other causes of systemic inflammatory responses [5], highlighting the urgent need to identify more reliable and powerful biomarkers for sepsis.

During septic shock, circulation fails to provide organs, such as the lungs and kidneys, with sufficient blood flow to meet the tissue’s metabolic needs, thus impairing overall cellular metabolism and function and ultimately leading to organ dysfunction and failure [1]. Because of insufficient supply of nutrients, many pathways are perturbed during sepsis, including inflammatory, neural, metabolic, and bioenergetic pathways. A common feature of these perturbed pathways is the excessive generation of reactive oxygen species (ROS), in which ROS can interfere with multiple signaling pathways, alter gene expression, and react with various biological molecules that can have deleterious functional outcomes [6]. Relatedly, sepsis is characterized by increased circulation of ROS species, indicating an overall state of dysfunction and oxidative stress [7], further implicating ROS in the pathogenesis of sepsis. Therefore, the objective of this study was to evaluate whether ROS-related genes can be used as a tool to predict sepsis survival. In this study, we analyzed two Gene Expression Omnibus (GEO) datasets from whole blood samples of septic patients and created an ROS-related gene signature. By analyzing the gene expression data, we found a 37-gene molecular signature that can predict survival of sepsis patients. These results suggest that peripheral blood gene expression data can be used to predict the survival of sepsis patients by assessing the free-radical state of patients. Recent advances in sepsis research recognize and appreciate that many pathways are perturbed during sepsis development [8,9,10]. Regardless, immune dysregulation undoubtably remains a vital factor in the pathology of sepsis [11,12,13]. Because of this, we concluded our study by evaluating the predicted immune cell profile in our low and high sepsis survival scores and found that the predicted proportion of immune cells does indicate a delineation of immune dysregulation between the scores.

## 2. Results

**Figure 1:**
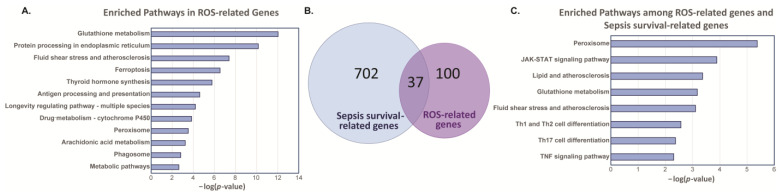
ROS-related genes and Sepsis Survival genes reveal 37-gene signature. (**A**) KEGG analysis was used to identify pathways enriched with ROS-related genes. (**B**) Comparison of differentially expressed genes between sepsis survival group compared to ROS-related genes reveals 37 common genes. (**C**) KEGG analysis was used to identify pathways enriched with 37-gene signature.

From the Molecular Signatures Database (MSigDB), we obtained a list of 137 genes that are related to ROS regulatory pathways (Supplementary Table S1). We then utilized Kyoto Encyclopedia of Genes and Genomes (KEGG) pathway analysis to assess the pathways related to ROS-related genes. We found that glutathione metabolism, endoplasmic reticulum protein processing, fluid shear stress, ferroptosis, and other pathways are enriched with the ROS-related genes (Figure 1A). To explore if these ROS-related genes/pathways are also involved in higher rates of septic mortality, we compared these ROS-related genes with sepsis survival-related genes. We have previously published the analysis of two human peripheral blood mononuclear cell datasets in ArrayExpress (E-MTAB-4421 and E-MTAB-4451) and showed the differentially expressed genes (DEGs) between survivors and non-survivors [14] (Supplementary Table S2). Using these DEGs in the sepsis survival group compared to the ROS-related genes, we found 37 common genes (Figure 1B). KEGG pathway analysis found that these 37 genes are enriched in the following pathways: peroxisome, JAK-STAT, lipid, glutathione metabolism, fluid shear stress, T-cell differentiation, and TNF signaling (Figure 1C).

**Figure 2:**
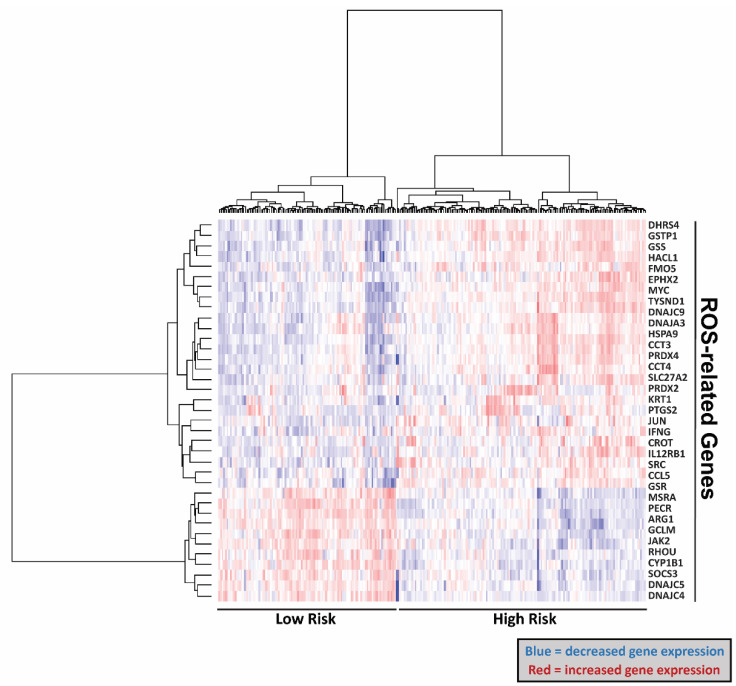
Heatmap of 37-gene signature expression in the discovery cohort. Heatmap of 37-gene signature expression reveals expression patterns between low and high-risk sepsis groups.

**Figure 3:**
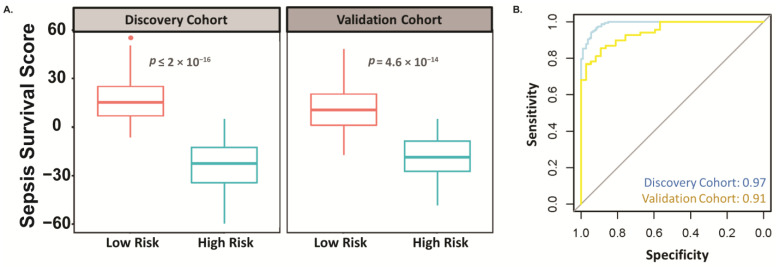
The 37-gene signature-based sepsis risk score differentiates sepsis high-risk patients from low-risk patients in both the discovery and validation cohort. (**A**) Box plot of risk scores in low and high-risk sepsis patients in both the discovery and validation cohorts. (**B**) ROC curves of the gene signature in distinguishing low and high-risk sepsis patients.

**Figure 4:**
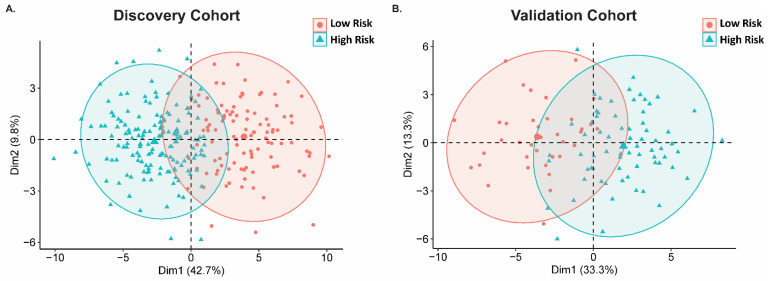
PCA plot of 37-gene signature in both Discovery and Validation Cohorts. Principal component analysis (PCA) on our 37-gene expression model was performed to reduce dimensionality and assess the similarity between each individual sample. In both the Discovery (**A**) and Validation (**B**) cohorts, the PCA showed that the 37 gene signature can entirely or mostly differentiate the high-risk sepsis patients from the low-risk sepsis patients. This PCA analysis represents 46.6–52.5% of the variable expression data.

Next, we generated a heatmap that separated the expression levels of the ROS-related genes between high risk and low sepsis risk groups, where the high sepsis risk group indicates a worse prognosis. In doing so, we found that this signature can distinguish the high and low risk groups (Figure 2). Specifically, our gene signatures identified 26 genes that are upregulated and 11 genes that are downregulated in the sepsis low-risk cohort (Table 1). We then used a sepsis survival score formula, which we have already published, to evaluate the probability of a clinical outcome in each of the sepsis patients in the transcriptome data we used in this study [14]. This sepsis survival score formula utilizes a linear combination of the included genes in the ROS-gene signature to the individual patient’s gene expression. A lower sepsis survival score predicts a worse prognosis and survival rate. Using this methodology, we found that the predicted sepsis survival score for the low sepsis risk group was significantly lower than the corresponding survival scores for the high sepsis risk group, in both the discovery (*p* < 2 × 10^−16^) and validation (*p* = 4.6 × 10^−14^) cohorts **(**Figure 3A). Moreover, the area under the receiver operating characteristic curve (AUC) value suggests that this gene signature has high sensitivity and specificity in both the discovery (AUC: 0.97) and validation cohort (AUC: 0.91) (Figure 3B). We then performed principal component analysis (PCA) on our 37-gene expression model to reduce dimensionality and assess the similarity between each individual sample. In both the discovery and validation cohorts, the PCA showed that the 37 gene signature can entirely or mostly differentiate the high-risk sepsis patients from the low-risk sepsis patients (Figure 4).

**Figure 5:**
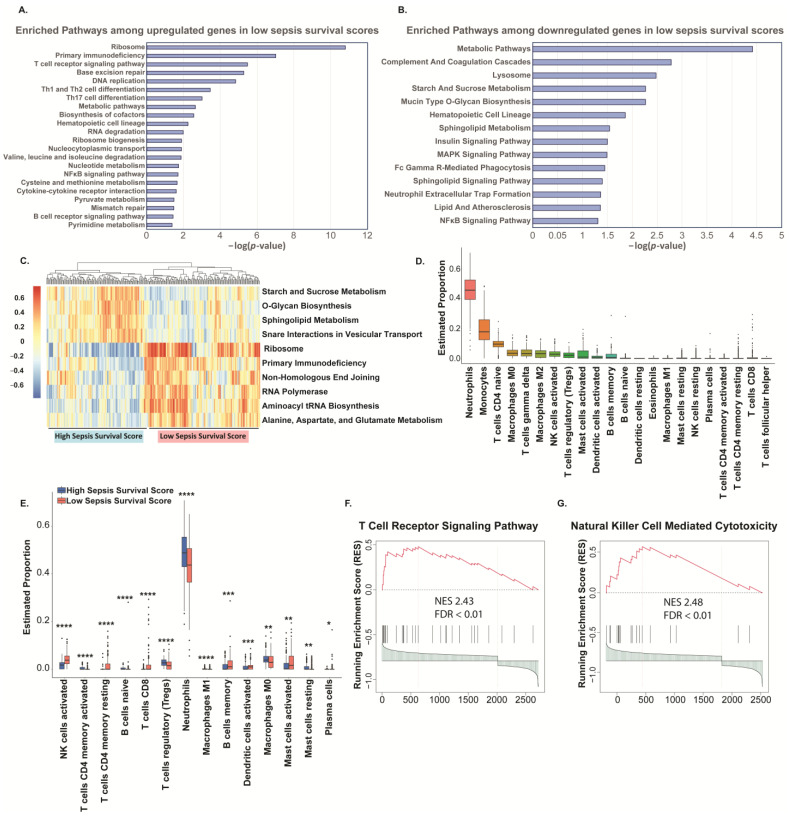
Immune Profiling of PBMCs in sepsis low and high-risk groups. KEGG analysis for (**A**) up-regulated or (**B**) down-regulated DEGs in low sepsis survival scores (**C**) Gene set variation analysis heatmap reveals the variations of pathways between high and low sepsis survival scores. (**D**) Using CIBERSORT, immune cell proportions in PBMCs were estimated. (**E**) In patients with high sepsis survival scores, neutrophils are predicted to be increased, while CD8+ T and NK cells are predicted to be decreased. * *p* < 0.05, ** *p* < 0.01, *** *p* < 0.001, **** *p* < 0.0001. (**F**,**G**) T and NK cell pathways were significantly decreased in patients with high sepsis survival scores. NES: normalized enrichment score.

We then performed KEGG pathway analysis on the low sepsis survival score group’s DEGs and found that 23 pathways are enriched with the upregulated genes (Figure 5A) and 14 pathways are enriched with the downregulated genes (Figure 5B). Additionally, we compiled a pathways heatmap to assess the gene variation of these pathways between sepsis high and low risk (Figure 5C) and see a clear distinction between the two groups. Comparing these enriched pathways revealed multiple immune cell pathways. For example, the upregulated genes are enriched in T and B-receptor signaling, Th1, Th2, and Th17 differentiation and the enriched pathways among downregulated genes in low sepsis survival scores include neutrophil extracellular trap formation. Additionally, both upregulated and downregulated genes are enriched among the hematopoietic cell lineage pathway. Moreover, it is known that the immune system plays critical roles in the progression of sepsis, although the molecular mechanisms underlying immune dysregulation within sepsis are still poorly understood [11,12]; to remedy this, we investigated the proportion of immune cells in peripheral blood mononuclear cell (PBMC) to delineate immune dysregulation between sepsis high and low risk groups. Using CIBERSORT, we established a reference for estimated proportions of immune cells in PBMC (Figure 5D). We then compared the predicted immune cell distribution between the high and low risk groups and found that levels of neutrophils are increased in the high-risk sepsis group, while CD8+ T cells and natural killer cells are decreased (Figure 5E). The T and natural killer cell pathways had normalized enrichment scores greater than 2.4 (Figure 5F,G).

**Figure 6:**
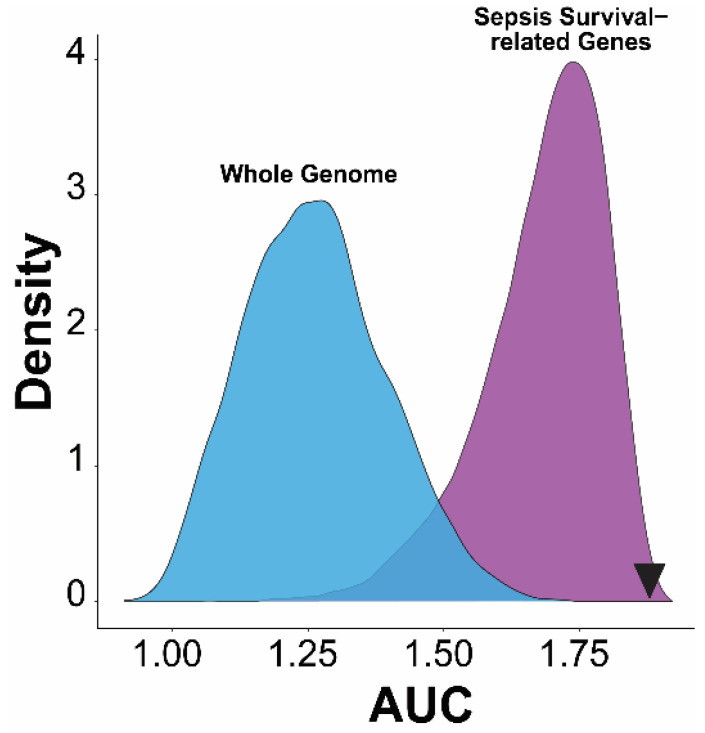
Density distribution plot of random gene signatures from the whole genome genes and only sepsis survival-related genes. Random gene signatures (37 genes) were selected from whole genome or sepsis survival-related genes and the performance of survival prediction was calculated as AUC (sum of AUC in discovery and validation cohorts). Compared with 10,000 randomly selected gene signatures, the 37-ROS-gene signature (AUC = 1.88, black inverted triangle) performs better than 99% of the gene signatures in both groups.

Lastly, to validate that our 37-gene signature performs significantly better than other combinations of gene signatures, we compared the diagnostic performance of this 37-gene signature with random gene signatures from the whole genome genes or only sepsis survival-related genes (Figure 6) [15,16]. The sum of the 37-gene signature-based AUC value (1.88) in the discovery and validation cohort is better than over 95% of random gene signatures (10,000 randomly times selected 37 genes) selected from whole genome or sepsis survival-related genes. This robust quality control data confirmed the significance of the prognostic power of this ROS-gene signature, and highly suggests that it was not population-specific.

## 3. Discussion

There are currently no therapeutics available to treat sepsis. Only early recognition and treatment has been shown to improve patient outcome [17,18]. Therefore, early diagnosis and action is critical for reducing mortalities from sepsis. Success in lowering sepsis mortality requires that clinicians must repeatedly draw blood cultures, monitor serum lactic acid levels, quickly administer antibiotics and aggressive fluid therapy, repeatedly assess fluid responsiveness, and possibly administer vasopressors [19,20]. Unfortunately, this protocol is intensive and only successful if performed during early signs of sepsis, meaning some patients are placed on this protocol too late as identifying early signs of sepsis remains challenging. Moreover, identifying specific and sensitive biomarkers for patients at high-risk for sepsis mortality is critical to reduce the high rates of morbidity and mortality of sepsis patients. If able to successfully identify those most at risk, these patients can be placed on treatments faster and be monitored more frequently for signs of sepsis progression to septic shock and organ failure.

Currently, prognostic biomarkers for sepsis survival have low sensitivity and specificity. For example, one multicenter prospective trial assessed lactate and the soluble form of plasminogen activator receptor as prognostic biomarkers for sepsis survival and found that these biomarkers only exhibit an AUC value of 0.70 and 0.77, respectively [21], highlighting that these assessments fail to recognize some severe sepsis cases. Moreover, an observational study assessed the following biomarkers as an indicator of sepsis-survival in sepsis patients admitted to the ICU: procalcitonin (AUC: 0.57), C-reactive protein (AUC: 0.51), interleukin-6 (AUC: 0.69), and monocyte chemotactic protein 1 (AUC: 0.64) [22]. These low AUC values highlight the urgent need to identify better prognostic biomarkers. Here, our ROS-gene signature appears to have AUC value above 0.90, supporting further efforts to evaluate our ROS-gene signature in sepsis prognosis (Figure 3B). To condense this large dataset for easy visualization, we performed a PCA analysis (Figure 4). It is important to note that this PCA analysis only represents 46.6–52.5% of the variable expression data, therefore the ability to mostly differentiate especially using methodologies that do not reduce dimensionality will likely separate these cohorts even more, suggesting that our gene signature does indeed separate low and high-risk patients relatively well.

The aforementioned prognostic biomarkers may be limited as they are primarily markers of inflammation. Although sepsis was classically considered to be primarily an overactive inflammatory response, recent advances support that sepsis involves dysfunction in the entire regulation of the cell, including alterations in cell metabolism [23]. Support for sepsis being more than an inflammatory disease mounted so much so that the Third International Consensus Definitions for Sepsis and Septic Shock changed the definition of sepsis to specifically state dysregulated host response to infection rather than dysregulated inflammation [1]. Sepsis is known to promote pro-oxidant redox states in sepsis patients, and this oxidant state results in excessive ROS generation which can affect a variety of cellular processes, such as metabolism and mitochondrial function [24,25,26].

Unfortunately, the rather unstable nature of ROS make clinical detection difficult and existing assays are prone to misinterpretation [27]. This means that there are conflicting reports in the literature about direct evidence for ROS in sepsis. For example, a study found no difference in ROS levels in serum of septic patients admitted to the ICU within 12-h compared to healthy volunteers [28]. Conversely, rats subjected to cecal puncture, a sepsis model, have increased levels of serum ROS, collected by cardiac puncture [29]. This highlights the complexity of studying mechanisms of disease, as there are many uncontrollable factors that are involved in the clinical level. For example, the rat model does not fully recapitulate the clinical progression of sepsis. Specifically, once patients enter the hospital they receive medical attention, including fluid and antibiotic administration, while the rats remain untreated. These interventions may occlude ROS detection. Moreover, serum collection from the rats were mixed oxygen blood from the heart, while patient’s serum is collected from veins which transport deoxygenated bloods. Despite these complications, evidence for the involvement of ROS in sepsis severity is still strong [6,30,31,32]. For instance, septic animals exhibit increased markers of oxidative damage models [33] and septic patients with less antioxidant capacity are associated with higher 30-day mortality [34]. Moreover, patients with Sequential Organ Failure Assessment (SOFA) scores above 7 have higher levels of serum ROS [35]. Although antioxidant therapies continually fail to improve septic outcomes in patients [36,37,38,39,40,41], this does not diminish the impact that ROS has on sepsis. Specifically, merely targeting ROS is an over simplistic approach to a complicated etiology. ROS themselves are not the problem, but rather, the effect they have on many other pathways, functional outcomes, and on gene expression [6,42,43,44]. That is why we investigated alterations in ROS-related genes to identify those with a more robust ROS response system who are subsequently more at risk for death. Additionally, our gene signature offers a prognostic tool with less ambiguity than current ROS assays and gives insight on the detrimental effects of ROS rather than the concentration of the species alone. Moreover, ROS has implications in many cellular processes that may give more insight into a patient’s overall state than assessing inflammation only. Therefore, coupling our gene signature with other markers of severe sepsis, such as high SOFA scores, can further increase the chance a physician can identify patients at risk for more severe sepsis outcomes.

Although other factors than inflammation play a critical in sepsis progression, it is undisputed that immune dysregulation is a vital component of sepsis pathology [11,12]. Because ROS are implicated in both contributing to and resulting from immune dysfunction [45], we aimed to gain insight on the immune dysregulation between sepsis high and low risk groups. We found that proportion of immune cells in peripheral blood mononuclear cell (PBMC) do indicate a delineation of immune dysregulation between sepsis high and low risk groups (Figure 5D–G). Specifically, we report a reduction of CD8+ T and natural killer (NK) cells in PBMC using CIBERSORT [46] to predict immune cell distribution. Our prediction matches other reports, for example, patients with severe cases of SARS-COV-2-induced acute respiratory syndrome have reduced levels of CD8+ T and NK cells, measured using flow cytometry [47]. CD8+ T and NK cells are cytotoxic effector cells in the immune system, with direct capabilities of killing infected cells or tumors [48]. Importantly, these cells appear to be sensitive to ROS. For example, prolonged exposure to ROS has been shown to inhibit phosphorylation of nuclear factor kappa-light-chain-enhancer of activated B cells (NF-κB), ultimately downregulating T cell activity [49]. Therefore, in high oxidative stress environments, the ability of these cells to clear infected cells may be decreased, further contributing to sepsis progression.

The clinical application of this particular blood genomic biomarker in daily practice presents potential advantages and drawbacks. This test might offer rapid access to patient data, potentially reducing turnaround time (TAT) for physicians and enabling more timely interventions in managing the “golden-hour” sepsis continuum with enhanced personalized treatment strategies by identifying markers associated with susceptibility or therapeutic outcomes. However, challenges such as the availability of comprehensive databases, interpretation of complex genomic/genetic information, and integration into clinical decision-making processes remain significant hurdles. A limitation of this study is that the peripheral blood mononuclear cells were collected up to five days after ICU admission and only one sample was taken. For better diagnostic assessment, immediate collection of repeated samples would be the most beneficial to monitor the progression of the potential biomarker. These datasets do not include the day the samples were collected per patient, which may be a confounding factor as samples collected at day 1 may differ from those collected at day 5. Therefore, to validate this ROS-gene-signature as a prognostic biomarker, datasets from peripheral blood mononuclear cells collected after first signs of sepsis or immediately after ICU admission. Moreover, assessment of the ROS-gene signature in patients with infections who eventually do or do not develop sepsis would provide valuable insight, as if this gene signature is able to identify those prone to developing sepsis, intervention can be introduced immediately and hopefully reduces the number of patients who die due to sepsis.

Of importance to note, the creation of a stratification system based on a person’s symptoms, lab values, comorbidities, and other factors would allow for the selection of biomarkers with better predictive prognostic value for that individual patient. For example, pro-adrenomedullin (MR-proADM) was originally predicted to have an AUC of 0.81 [50,51]. Unfortunately, later studies found that the AUC was lower, with a new AUC value of 0.61. However, these researchers found that comorbidities, such as chronic obstructive pulmonary disorder (COPD) confounded their prediction, as these patients have higher MR-proADM regardless of sepsis infection status. When COPD patients were removed from the analysis, MR-proADM AUC was increased to 0.69 [52]. Although 0.69 indicates a low predictive value, it highlights the concept that one biomarker will likely never be sufficient to predict outcomes in a disease as vast as sepsis. Further evidence of this is that presepsin has a predictive AUC value of 0.925 in children [53], while in adults, the AUC value drops to 0.656 [54]. Our ROS gene signature identifies genes that are altered during a variety of known sepsis-related pathway and thus presents an opportunity to screen for sepsis risk in a variety of patients whose individual etiology of sepsis differs from one another but results in an overall increase expression of ROS-related genes. Our gene signature is especially valuable if medical history is unknown.

## 4. Materials and Methods

### 4.1. Transcriptomic Datasets and Sepsis Survival-Related Genes

We previously searched through ArrayExpress and Gene Expression Omnibus (GEO) databases and identified two human peripheral blood mononuclear cell (PBMC) datasets in ArrayExpress (E-MTAB-4421 and E-MTAB-4451) that matched our search criteria of having survival information and greater than 100 samples [14]. E-MTAB-4421 (265 sepsis patients) was used as the discovery cohort and E-MTAB-4451 (includes 106 sepsis-survival patients) was used as the validation cohort. Samples from adult patients (>18 years) were collected up to 5 days after admission to intensive care unit (ICU) for severe sepsis due to community acquired pneumonia. Survival was assessed after 28 days of ICU admission. Differentially expressed genes were defined as significant if the fold change was greater than 1.5 and the false discovery rate was less than 5%. To identify sepsis survival-related genes, the R packages “limma” and “gcrma” (versions 3.13) were used to detect differentially expressed genes between sepsis high risk (SHR) and sepsis low risk (SLR) groups in the discovery cohort (E-MTAB-4421).

### 4.2. Sepsis Survival Score

To calculate the sepsis survival score, a linear combination of gene expression values and corresponding weight values in the ROS-related gene signature. The sepsis survival score formula used is indicated below, where n represents the genes counted in the ROS-related gene signature for each dataset, W_i_ shows the weighted value of each gene, e_i_ indicates the expression level of each gene, and μ_i_ and S_i_ are the mean and standard deviation values for the corresponding gene among the whole sample. To stratify our scores into two groups, we first determined the mean of the sepsis survival score within our data set. Values above the mean were classified into the high sepsis survival score cohort, while the values below the mean were classified into the low sepsis survival score cohort.

$$sepsis\;survival\;score=\sum_{i=1}^{n}W_{i}\left(\frac{e_{i}-\mu_{i}}{S_{i}}\right)$$

### 4.3. Enrichment Analysis

In this study, we used the database for annotation, visualization, and integrated discovery (DAVID, version 6.8, https://david.ncifcrf.gov/tools.jsp, accessed on 21 September 2023) to perform enrichment analysis among ROS-related Genes and/or Sepsis-survival genes. DAVID is a biological and functional annotation database. Here, we utilized DAVID for interpretation of gene/signaling pathway interactions for the genes in our lists. Adjusted *p*-values < 0.05 were considered significant.

### 4.4. CIBERSORT

To confirm that our risk-scoring system could accurately represent the status of the immune system, we estimated the proportions of immune cells in the sepsis datasets and evaluated if there were different immune cell proportions between our sepsis low- and high-risk groups. We used the R package CIBERSORT (version 0.1.0) [46] to estimate the abundances of major immune cell types using gene expression data in the blood samples from the discovery cohort. The gene signature matrix (LM22) provided by CIBERSORT (https://cibersortx.stanford.edu, accessed on 21 September 2023), which contains 547 genes and all major immune cell types, was used as input for reference gene expression signatures. The CIBERSORT scales each cellular fraction to a score that represents each cell type’s proportion. The proportions of cell types were extracted and visualized using the R package ggpubr (version 0.6.0).

### 4.5. Running Enrichment Score

The gene expression matrix of the discovery cohort and 186 canonical pathway gene sets derived from the KEGG pathway were used as input for gene set enrichment analysis (GSEA). The R package GSEA_R (version 1.2) was utilized to perform the enrichment analysis, and the running parameters were set to default.

### 4.6. Statistical Analysis

Statistical calculations were performed using the computer language, R. The R packages ade4 (Version: 1.7-22) and pROC (Version: 1.18.5) were used to create the PCA plots and ROC curves. Values with false discovery rates (FDR) < 0.05 were considered to be significant.

## 5. Conclusions

Our results indicate that the generated ROS-gene signature has clinical relevancy and should be evaluated further as a prognostic marker for sepsis survival and to identify patients most at-risk for mortality.

## Figures and Tables

**Table 1:** List of genes in free radical associated gene signature.

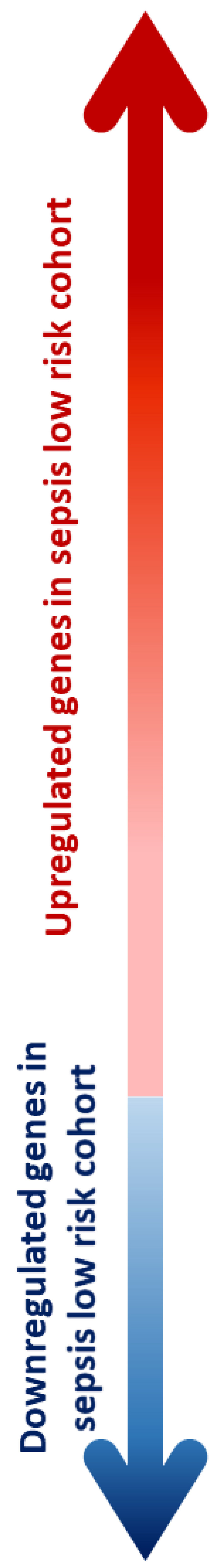	**Gene Symbol**	**Gene Name**	**Log (Fold Change)**	**Weight**
	KRT1	Keratin 1	1.39649507	1
	BCL2	BCL2 Apoptosis Regulator)	1.21684097	1
	TYSND1	Trypsin Like Peroxisomal Matrix Peptidase 1	1.028826515	1
	DHRS4	Dehydrogenase/Reductase 4	1.023064207	1
	EPHX2	Epoxide Hydrolase 2	0.998809446	1
	IFNG	Interferon Gamma	0.990314958	1
	CCL5	Chemokine (C-C motif) ligand 5	0.978813828	1
	MYC	Myc myelocytomatosis oncogen	0.942214034	1
	SRC	SRC proto-oncogene, non-receptor tyrosine kinase	0.919788893	1
	PRDX4	Peroxiredoxin 4	0.905019227	1
	GSS	glutathione synthetase	0.868091871	1
	HACL1	2-Hydroxyacyl-CoA Lyase 1	0.806398241	1
	DNAJA3	DnaJ Heat Shock Protein Family (Hsp40) Member A3	0.796338833	1
	GSTP1	Glutathione S-Transferase Pi 1	0.718004102	1
	PRDX2	peroxiredoxin 2 (PRDX2)	0.709937194	1
	PTGS2	Prostaglandin-Endoperoxide Synthase 2	0.704762302	1
	HSPD1	60 kDa heat shock protein, mitochondrial	0.68481934	1
	FMO5	Flavin Containing Dimethylaniline Monoxygenase 5	0.677398254	1
	DNAJC9	DnaJ Heat Shock Protein Family (Hsp40) Member C9	0.676712675	1
	CCT4	Chaperonin Containing TCP1 Subunit 4	0.676162928	1
	IL12RB1	Interleukin 12 Receptor Subunit Beta 1	0.634905263	1
	HSPA9	Heat Shock Protein Family A (Hsp70) Member 9	0.627851503	1
	CROT	Peroxisomal carnitine O-octanoyltransferase	0.625008985	1
	CCT3	Chaperonin containing TCP1 subunit 3 (CCT3)	0.62440571	1
	JUN	Jun Proto-Oncogene, AP-1 Transcription Factor Subunit	0.61663915	1
	SLC27A2	Solute Carrier Family 27 Member 2	0.595969275	1
	GSR	Glutathione-Disulfide Reductase	−0.613066934	−1
	JAK2	Janus Kinase 2	−0.639496536	−1
	DNAJC5	DnaJ Heat Shock Protein Family (Hsp40) Member C5	−0.682604763	−1
	DNAJC4	DnaJ Heat Shock Protein Family (Hsp40) Member C4	−0.746858619	−1
	RHOU	Rho-related GTP-binding protein RhoU	−0.825335335	−1
	GCLM	glutamate-cysteine ligase, modifier subunit	−0.904446168	−1
	MSRA	Methionine Sulfoxide Reductase A	−0.984304987	−1
	SOCS3	Suppressor of cytokine signaling 3	−1.259719259	−1
	PECR	Peroxisomal Trans-2-Enoyl-CoA Reductase	−1.476257053	−1
	CYP1B1	Cytochrome P450 Family 1 Subfamily B Member 1	−1.517326189	−1
	ARG1	Arginase 1	−1.546803504	−1

## Data Availability

All original research data supporting reported results can be available upon request.

## Supplementary Materials

The following supporting information can be downloaded at: https://www.mdpi.com/article/10.3390/ijms25084574/s1.
