# Supplementary material for: Free Radical–Associated Gene Signature Predicts Survival in Sepsis Patients
Source: Int J Mol Sci. 2024 Apr 22;25(8):4574. doi: 10.3390/ijms25084574 (PMC11049877; doi:10.3390/ijms25084574)
Supplement: Supplementary file 1 [file ijms-25-04574-s001.zip › Supplemental Table S1.pdf]

Supplemental Table S1: ROS-related gene list.

ROS-related Gene List	
**Gene Symbol**	**Gene Name**
ALB	Albumin
ALOX12	Arachidonate 12-lipoxygenase
APOE	Apolipoprotein E
ATOX1	ATX1 antioxidant protein 1 homolog
BAG1	BCL2-associated athanogene
BCL2	B-cell CLL/lymphoma 2
BNIP3	BCL2/adenovirus E1B 19kDa interacting protein 3
CALR	Calreticulin
CANX	Calnexin
CAT	Catalase
CCL5	Chemokine (C-C motif) ligand 5
CCS	Copper chaperone for superoxide dismutase
CCT2	Chaperonin containing TCP1, subunit 2 (beta)
CCT3	Chaperonin containing TCP1, subunit 3 (gamma)
CCT4	Chaperonin containing TCP1, subunit 4 (delta)
CCT5	Chaperonin containing TCP1, subunit 5 (epsilon)
CCT7	Chaperonin containing TCP1, subunit 7 (eta)
CCT8	Chaperonin containing TCP1, subunit 8 (theta)
CES1	Carboxylesterase 1 (monocyte/macrophage serine esterase 1)
CSDE1	Cold shock domain containing E1, RNA-binding
CYBA	Cytochrome b-245, alpha polypeptide
CYBB	Cytochrome b-245, beta polypeptide (chronic granulomatous disease)
CYP11A1	Cytochrome P450, family 11, subfamily A, polypeptide 1
CYP11B2	Cytochrome P450, family 11, subfamily B, polypeptide 2
CYP1A1	Cytochrome P450, family 1, subfamily A, polypeptide 1
CYP1B1	Cytochrome P450, family 1, subfamily B, polypeptide 1
CYP2C19	Cytochrome P450, family 2, subfamily C, polypeptide 19
CYP2D6	Cytochrome P450, family 2, subfamily D, polypeptide 6
CYP2F1	Cytochrome P450, family 2, subfamily F, polypeptide 1
DHCR24	24-dehydrocholesterol reductase
DNAJA1	DnaJ (Hsp40) homolog, subfamily A, member 1
DNAJA2	DnaJ (Hsp40) homolog, subfamily A, member 2
DNAJA3	DnaJ (Hsp40) homolog, subfamily A, member 3
DNAJA4	DnaJ (Hsp40) homolog, subfamily A, member 4
DNAJB1	DnaJ (Hsp40) homolog, subfamily B, member 1
DNAJB11	DnaJ (Hsp40) homolog, subfamily B, member 11
DNAJB12	DnaJ (Hsp40) homolog, subfamily B, member 12
DNAJB2	DnaJ (Hsp40) homolog, subfamily B, member 2
DNAJB4	DnaJ (Hsp40) homolog, subfamily B, member 4
DNAJB5	DnaJ (Hsp40) homolog, subfamily B, member 5
DNAJB6	DnaJ (Hsp40) homolog, subfamily B, member 6
DNAJB9	DnaJ (Hsp40) homolog, subfamily B, member 9
DNAJC1	DnaJ (Hsp40) homolog, subfamily C, member 1
DNAJC4	DnaJ (Hsp40) homolog, subfamily C, member 4
DNAJC5	DnaJ (Hsp40) homolog, subfamily C, member 5
DNAJC7	DnaJ (Hsp40) homolog, subfamily C, member 7
DNAJC8	DnaJ (Hsp40) homolog, subfamily C, member 8
DNAJC9	DnaJ (Hsp40) homolog, subfamily C, member 9
DUOX1	Dual oxidase 1
DUSP1	Dual specificity phosphatase 1
EPHX2	Epoxide hydrolase 2
EPX	Eosinophil peroxidase
FMO4	Flavin containing monooxygenase 4
FMO5	Flavin containing monooxygenase 5
FOS	v-fos FBJ murine osteosarcoma viral oncogene homolog
FOXM1	Forkhead box M1
FTH1	Ferritin, heavy polypeptide 1
GCLC	Glutamate-cysteine ligase, catalytic subunit
GCLM	Glutamate-cysteine ligase, modifier subunit
GLRX2	Glutaredoxin 2
GPR156	G protein-coupled receptor 156
GPX1	Glutathione peroxidase 1
GPX3	Glutathione peroxidase 3
GPX4	Glutathione peroxidase 4
GPX6	Glutathione peroxidase 6
GPX7	Glutathione peroxidase 7
GSR	Glutathione reductase
GSS	Glutathione synthetase
GSTA5	Glutathione S-transferase A5
GSTP1	Glutathione S-transferase pi
GSTZ1	Glutathione transferase zeta 1
GTF2I	General transcription factor II, i
HIF1A	Hypoxia-inducible factor 1, alpha subunit
HIP2	Huntingtin interacting protein 2
HMOX1	Heme oxygenase (decycling) 1
HMOX2	Heme oxygenase (decycling) 2
HOPX	HOP homeobox
HSPA1A	Heat shock 70kDa protein 1A
HSPA1L	Heat shock 70kDa protein 1-like
HSPA2	Heat shock 70kDa protein 2
HSPA4	Heat shock 70kDa protein 4
HSPA5	Heat shock 70kDa protein 5
HSPA8	Heat shock 70kDa protein 8
HSPA9	Heat shock 70kDa protein 9
HSPB1	Heat shock 27kDa protein 1
HSPB2	Heat shock 27kDa protein 2
HSPD1	Heat shock 60kDa protein 1
HSPE1	Heat shock 10kDa protein 1
JUNB	Jun B proto-oncogene
KEAP1	Kelch-like ECH-associated protein 1
KRT1	Keratin 1
MB	Myoglobin
MGST3	Microsomal glutathione S-transferase 3
MPO	Myeloperoxidase
MPV17	MpV17 mitochondrial inner membrane protein
MSRA	Methionine sulfoxide reductase A
MTLÂ 5	Metallothionein-like 5
NCF1	Neutrophil cytosolic factor 1
NCF2	Neutrophil cytosolic factor 2
NOX4	NADPH oxidase 4
NQO1	NAD(P)H dehydrogenase, quinone 1
NUDT1	Nudix (nucleoside diphosphate linked moiety X)-type motif 1
OXR1	Oxidation resistance 1
OXSR1	Oxidative-stress responsive 1
PDLIM1	PDZ and LIM domain 1 (elfin)
PNKP	Polynucleotide kinase 3â€²-phosphatase
PON1	Paraoxonase 1
PPID	Peptidylprolyl isomerase D (cyclophilin D)
PRDX1	Peroxiredoxin 1
PRDX2	Peroxiredoxin 2
PRDX3	Peroxiredoxin 3
PRDX4	Peroxiredoxin 4
PRDX5	Peroxiredoxin 5
PRDX6	Peroxiredoxin 6
PREX1	Phosphatidylinositol 3,4,5-trisphosphate-dependent RAC exchanger 1
PRG3	Proteoglycan 3
PRNP	Prion protein (p27â€“30)
PTGS1	Prostaglandin-endoperoxide synthase 1
PTGS2	Prostaglandin-endoperoxide synthase 2
RNF7	Ring finger protein 7
SCARA3	Scavenger receptor class A, member 3
SELS	Selenoprotein S
SERPINH1	Serpin peptidase inhibitor, clade H (heat shock protein 47), member 1
SFTPD	Surfactant, pulmonary-associated protein D
SGK2	Serum/glucocorticoid regulated kinase 2
SIRT2	Sirtuin 2
SOD1	Superoxide dismutase 1
SOD2	Superoxide dismutase 2
SQSTM1	Sequestosome 1
SRXN1	Sulfiredoxin 1
ST13	Suppression of tumorigenicity 13
STK25	Serine/threonine kinase 25
TCP1	T-complex 1
TTN	Titin
TXNRD1	Thioredoxin reductase 1
TXNRD2	Thioredoxin reductase 2
UCP2	Uncoupling protein 2
