# Supplementary material for: Free Radical–Associated Gene Signature Predicts Survival in Sepsis Patients
Source: Int J Mol Sci. 2024 Apr 22;25(8):4574. doi: 10.3390/ijms25084574 (PMC11049877; doi:10.3390/ijms25084574)
Supplement: Supplementary file 1 [file ijms-25-04574-s001.zip › Supplemental Table S2.pdf]

# Supplemental Table S2: Sepsis-survival related genes.

| ROS-related Gene List | | | |
|---|---|---|---|
| Gene Symbol | Gene Name | logFC | adj-p |
| FGFBP2 | fibroblast growth factor binding protein 2 | 2.21409787 | 6.96E-24 |
| TNFRSF25 | TNF receptor superfamily member 25 | 2.16760952 | 3.51E-37 |
| CD5 | CD5 molecule | 2.13358987 | 2.40E-42 |
| S1PR5 | sphingosine-1-phosphate receptor 5 | 2.11873122 | 4.53E-30 |
| C6orf190 | thymocyte selection associated | 2.09687165 | 4.61E-42 |
| ZAP70 | zeta chain of T cell receptor associated protein kinase 70 | 2.09231594 | 4.31E-39 |
| PVRIG | PVR related immunoglobulin domain containing | 2.03717016 | 4.91E-42 |
| BCL11B | BCL11 transcription factor B | 2.02797437 | 2.19E-34 |
| EOMES | eomesodermin | 2.00470494 | 3.15E-29 |
| CD6 | CD6 molecule | 1.99607843 | 5.24E-44 |
| CD27 | CD27 molecule | 1.99203512 | 1.04E-29 |
| FCER1A | Fc epsilon receptor Ia | 1.97530166 | 1.95E-20 |
| TCEA3 | transcription elongation factor A3 | 1.9710649 | 1.42E-30 |
| SBK1 | SH3 domain binding kinase 1 | 1.93412746 | 3.02E-43 |
| SKAP1 | src kinase associated phosphoprotein 1 | 1.92154118 | 6.00E-43 |
| UBASH3A | ubiquitin associated and SH3 domain containing A | 1.91069272 | 5.19E-35 |
| FLT3LG | fms related receptor tyrosine kinase 3 ligand | 1.90064791 | 1.17E-38 |
| GZMH | granzyme H | 1.89889382 | 9.66E-22 |
| CD3E | CD3 epsilon subunit of T-cell receptor complex | 1.8940005 | 1.13E-34 |
| CD4 | CD4 molecule | 1.89026653 | 3.37E-34 |
| CCR3 | C-C motif chemokine receptor 3 | 1.86615156 | 3.08E-15 |
| RORA | RAR related orphan receptor A | 1.8517576 | 4.51E-43 |
| CD160 | CD160 molecule | 1.8340921 | 4.58E-24 |
| D4S234E | neuronal vesicle trafficking associated 1 | 1.83339124 | 1.98E-29 |
| FCGBP | Fc gamma binding protein | 1.82061919 | 3.09E-29 |
| TRAF5 | TNF receptor associated factor 5 | 1.77965484 | 1.69E-38 |
| SLC25A38 | solute carrier family 25 member 38 | 1.77107373 | 3.77E-50 |
| GZMK | granzyme K | 1.77018351 | 2.07E-28 |
| RASGRP1 | RAS guanyl releasing protein 1 | 1.76774559 | 6.69E-42 |
| GPR162 | G protein-coupled receptor 162 | 1.7519198 | 2.22E-22 |
| FAM102A | estrogen-induced osteoclastogenesis regulator 1 | 1.75047508 | 1.44E-38 |
| GNLY | granulysin | 1.73917668 | 7.39E-21 |
| CD96 | CD96 molecule | 1.73169549 | 1.24E-36 |
| P2RY10 | P2Y receptor family member 10 | 1.72118381 | 9.16E-34 |
| HDC | histidine decarboxylase | 1.71824419 | 6.62E-18 |
| CD3D | CD3 delta subunit of T-cell receptor complex | 1.71499399 | 1.35E-35 |
| GDPD5 | glycerophosphodiester phosphodiesterase domain containing 5 | 1.71327959 | 3.92E-38 |
| MCOLN2 | mucolipin TRP cation channel 2 | 1.7024545 | 3.14E-31 |
| SLC25A23 | solute carrier family 25 member 23 | 1.70105034 | 1.82E-37 |
| PECI | enoyl-CoA delta isomerase 2 | 1.69985795 | 1.86E-42 |
| TLR7 | toll like receptor 7 | 1.69089876 | 1.31E-26 |
| ATP8B2 | ATPase phospholipid transporting 8B2 | 1.68652085 | 6.69E-36 |
| TSEN54 | tRNA splicing endonuclease subunit 54 | 1.68558135 | 2.03E-46 |
| CLIC3 | chloride intracellular channel 3 | 1.67655218 | 3.11E-20 |
| ZNHIT6 | zinc finger HIT-type containing 6 | 1.67072237 | 7.41E-50 |
| OAF | out at first homolog | 1.66801079 | 1.18E-32 |
| MME | membrane metalloendopeptidase | 1.66223671 | 6.95E-15 |
| HLA-DMB | major histocompatibility complex, class II, DM beta | 1.66222371 | 2.70E-33 |
| NOV | cellular communication network factor 3 | 1.65984408 | 1.73E-22 |
| GZMA | granzyme A | 1.65367098 | 2.41E-25 |
| KLHL3 | kelch like family member 3 | 1.6531521 | 9.13E-38 |
| C16orf30 | transmembrane protein 204 | 1.65084835 | 1.13E-33 |
| LGALS2 | galectin 2 | 1.64687982 | 3.98E-18 |
| SULF2 | sulfatase 2 | 1.64220916 | 1.89E-25 |
| FLJ14213 | proline rich 5 like | 1.63245609 | 4.40E-26 |
| TGFBR3 | transforming growth factor beta receptor 3 | 1.62461497 | 1.12E-36 |
| PRKDC | protein kinase, DNA-activated, catalytic subunit | 1.62338941 | 1.76E-20 |
| ITK | IL2 inducible T cell kinase | 1.61544488 | 1.66E-34 |
| NOC3L | NOC3 like DNA replication regulator | 1.61306989 | 1.25E-34 |
| RSAD1 | radical S-adenosyl methionine domain containing 1 | 1.61201737 | 6.49E-47 |
| CD247 | CD247 molecule | 1.60967392 | 4.75E-35 |
| CD8A | CD8 subunit alpha | 1.59870455 | 4.08E-23 |
| TBC1D9 | TBC1 domain family member 9 | 1.59091886 | 1.02E-24 |
| PTPRCAP | protein tyrosine phosphatase receptor type C associated protein | 1.58839282 | 3.43E-35 |
| NELF | NMDA receptor synaptonuclear signaling and neuronal migration factor | 1.58099767 | 7.64E-39 |
| TTC27 | tetratricopeptide repeat domain 27 | 1.57947471 | 5.36E-43 |
| TARBP1 | TAR (HIV-1) RNA binding protein 1 | 1.57550461 | 2.43E-38 |
| KLRG1 | killer cell lectin like receptor G1 | 1.57258893 | 1.31E-30 |
| MEF2C | myocyte enhancer factor 2C | 1.5695608 | 6.26E-32 |
| MBNL2 | muscleblind like splicing regulator 2 | 1.56769051 | 2.87E-33 |
| TCF3 | transcription factor 3 | 1.56242825 | 2.43E-39 |
| IL7R | interleukin 7 receptor | 1.55882094 | 5.06E-32 |
| C2orf89 | TraB domain containing 2A | 1.55869534 | 3.48E-30 |
